# Practical opportunities for microbiome analyses and bioinformatics in poultry processing

**DOI:** 10.1016/j.psj.2022.101787

**Published:** 2022-02-18

**Authors:** Steven C. Ricke, Dana K. Dittoe, Jessica A. Brown, Dale R. Thompson

**Affiliations:** ⁎ Meat Science and Animal Biologics Discovery Program, Department of Animal and Dairy Sciences, University of Wisconsin, Madison, WI 53706, USA; † Department of Computer Science and Computer Engineering, University of Arkansas, Fayetteville, AR 72701, USA

**Keywords:** poultry processing, microbiota, bioinformatics, microbiome, education

## Abstract

Poultry processing is undergoing changes both in operations as well as microbial methodologies. Traditionally, microbial data has been gathered through a series of culturing methods using liquid media and plating for isolation and enumeration. Both foodborne pathogens and nonpathogenic bacterial populations are estimated to assess food safety risks as well as the potential for spoilage. Bacterial loads from carcasses are important for estimating processing control and the effectiveness of antimicrobial applications. However, these culture-based approaches may only provide part of the microbial ecology landscape associated with chicken carcasses and the subsequent changes that occur in these populations during processing. Newer molecular-based approaches, such as 16S sequencing of the microbiota, offer a means to retrieve a more comprehensive microbial compositional profile. However, such approaches also result in large data sets which must be analyzed and interpreted. As more data is generated, this will require not only bioinformatic programs to process the data but appropriate educational forums to present the processed data to a broad audience.

## Introduction

Conversion of live poultry into various meat products is a complex process involving a multitude of steps before the final products reach retail markets. Numerous advances have been made over the years to increase poultry processing line speeds, improve uniformity, and decrease the environmental footprint. As these improvements and technical advancements are introduced, this adds to the complexity of poultry processing operations. In addition, depending on market demands, processing plants must accommodate different sizes of birds, variation in retail market destinations, and types of meat products including whole rotisserie carcasses, chicken parts, ground meat, and further processed products such as nuggets. As markets become more expansive with the emergence of consumer interest in organic, natural, and pasture-raised sources of poultry, this diversification will only expand. Developments in the application of automation technologies in processing environments add further complexity to poultry and meat processing (Chao et al., 2014). Unforeseen issues, such as the 2020 Covid-19 pandemic, have complicated processing plant operations from a labor and meat supply standpoint, with large-scale meat processors being particularly vulnerable to the spread of the disease (Taylor et al., 2020). The results of this pandemic may support an acceleration of the development of automation technology in the meat and poultry processing industry to retain and increase line speeds.

The microbial ecology of poultry processing environments can be relatively complex. There are several contributing factors to the microbial composition of a poultry carcass as it moves through the various processing steps. Not only do incoming birds harbor fecal and gastrointestinal microorganisms, but they also carry an additional bacterial load on their feathers, feet, and other extremities. As these birds are eviscerated, followed by additional stages of processing, they have the opportunity to come in contact with a wide range of environmental microorganisms and other sources of cross-contamination, which contribute to the overall microbial population of the carcass. The microbial composition of the contaminants on the carcass is somewhat variable and can consist of both pathogens and nonpathogens. Numerous culture and molecular-based methodologies have been developed for monitoring the presence of foodborne pathogens on carcasses, but less is known about the identities of the nonpathogenic portion of the poultry carcass microbial community. However, these nonpathogenic microorganisms are also important as their presence can lead to considerable economic losses upon their proliferation followed by spoilage and shortening of shelf life. Culture-based methods have been traditionally used to “biomap” these organisms through the processing environment (Feye et al., 2020a). The introduction of microbiome sequencing and bioinformatics offers the potential opportunity to identify individual members of these microbial communities, more precisely predict shelf life, and ultimately devise the appropriate mitigation strategies. In this review, the potential applications for microbiome analytics in poultry processing, along with the development of training and educational tools will be discussed.

## Poultry Processing and Microbial Contamination

**Figure 1:**
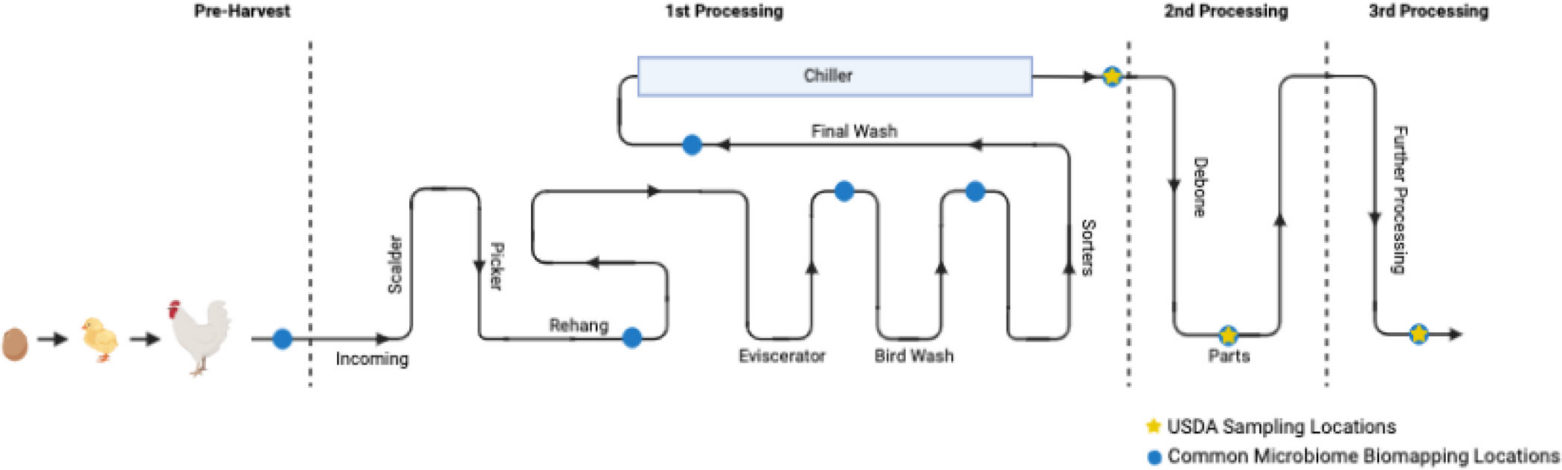
Illustration of the longitudinal pathway of a broiler from farm to fork. Common microbiome and biomapping sample locations, and official USDA sampling locations are labeled with a circle and star, respectively. Figure created with Biorender.com.

There are essentially 3 stages to commercial poultry processing (Figure 1). These stages consist of first, second, and further or third processing (Handley et al., 2015). The details associated with each of these steps have been described extensively in previous reviews, and only a brief overview will be covered in the current review (Owens et al., 2010; Handley et al., 2015; Blevins et al., 2017). Poultry processing operations categorized as first processing include all steps from receipt of live birds through slaughtering, defeathering, evisceration, washing, and chilling (Figure 1). Second processing occurs after chilling with the generation of parts from whole carcasses such as breasts, wings, thighs, among others, and this can also include steps such as deboning, skinning, seasoning, or injection of marinade (Owens et al., 2010; Handley et al., 2015). Ready-to-eat (RTE) poultry products, including nuggets, chicken patties, breaded tenders, and coated meat products produced via cutting, coating, and cooking processes are generated in the third processing stage (Owens et al., 2010; Handley et al., 2015).

Given the complexity of the multiple processing steps and wide range of retail destinations for poultry meat products exiting the poultry processing plant, microbial exposure and subsequent impact on the potential pathogen and nonpathogen contamination remain an ongoing concern. Several sources of microorganisms can contribute to carcass contamination, including fecal content on incoming birds as well as cross-contamination that occurs during processing (Handley et al., 2015). Cross-contamination can originate from processing plant environmental sources, bird-to-bird contact, and the accumulation of microorganisms on processing equipment. The formation of biofilms on equipment, as well as environmental niches throughout the processing plant, can contribute to the persistence of certain microorganisms and present challenges when cleaning and sanitizing plants and equipment. Since pathogens can contribute to contamination from these sources, the introduction of Hazard Analysis of Critical Control Points (**HACCP**) assessments to identify potential problematic food safety issues for each poultry meat product is important (Handley et al., 2015). The development of specific HACCP plans leads to the location of likely targets of microbial contamination and, in turn, the development of prevention strategies to mitigate food safety concerns (Handley et al., 2015). In conjunction with HACCP, the introduction of physical and chemical interventions represents a means for the poultry industry to reduce both pathogen and non-pathogen loads on carcasses as they progress through the processing plant. Likewise, the application of sanitizers to the processing plant equipment and environment is a critical step in reducing microbial contamination throughout the plant.

Therefore, it is not surprising that microbial levels and sanitation effectiveness are monitored to minimize microbial contamination during and after processing. Monitoring levels of microorganisms is focused on both the birds as well as the poultry processing plant environment. For verification of sanitation effectiveness in the poultry processing plant, results from swabs based on either adenosine triphosphate (**ATP**) or bacterial plate counts have been used for nonproduct and product contact surfaces (Mead, 1995; Downes and Ito, 2001; Forsythe, 2010; Cramer, 2013; Blevins et al., 2017). Swabs using ATP rely on the amount of relative light units emitted by ATP from the residues and biofilms on swabbed surfaces (Aycicek et al., 2006; Cramer, 2013; Blevins et al., 2017). Cotton-tipped swabs can be used for collecting samples for bacterial enumeration, with the swabs typically being streaked onto aerobic plate count (**APC**) agar and the outcome reported as colony forming units (**CFU**) per square inch or centimeter (Downes and Ito, 2001). Swabs based on ATP have the advantage of yielding results within several minutes vs. the 24 to 48 hours required for sufficient bacterial colonies to be visible for bacterial plate enumeration, but ATP results cannot differentiate between nonbacterial proteins and bacterial cells (Forsythe, 2010; Cramer, 2013; Shama and Malik, 2013; Blevins et al., 2017). This has been seen with other contact surfaces. For example, this was demonstrated with comparisons between bacterial swab data and ATP swabs on samples recovered from Formica tabletops in a dining food service facility (Masuku et al., 2012). In this study, the 2 sets of results were not comparable, but they concluded that the ATP approach offered a means for real-time assessment of hygienic status, although bacterial enumeration was still required for a reliable measure of cleanliness. For poultry processing, a similar conclusion can be drawn that ATP data may not directly relate to bacterial counts, but still can be used as an indicator that a particular surface possesses enough bacterial load to be detected by ATP and/or sufficient substrates to potentially support bacterial growth (Blevins et al., 2017).

For poultry carcass microbial composition, USDA Food Safety and Inspection Service (**USDA-FSIS**) has prescribed methodologies for sampling and microbial analyses. Poultry carcasses are directly removed from selected sites in the processing line and placed in sterile shaker bags, then rinsed for 1 min in a 400 mL solution of sterile, refrigerated Butterfield phosphate buffer with 2.0 mL of 20,000 parts per million (**ppm**) sodium thiosulfate as a neutralizing agent. The resulting rinsate solutions can be subsequently removed for microbiological analyses. For enumeration of total bacterial load, aliquots from each rinsate solution are typically plated onto APC Petrifilm (3M, 3M Center Bldg., St. Paul, MN) at local plant laboratories (AOAC, 2005). Samples to be processed for *Salmonella* and *Campylobacter* analyses are generally shipped to an off-site laboratory. Samples are tested for the presence of *Salmonella* with a commercial polymerase chain reaction (**PCR**) assay and quantitated by direct plating on tryptic soy agar plates with an XLT4 overlay, while *Campylobacter* spp. are enumerated by direct plating on selective agar plates (Chipley, 1987; Kang and Fung, 2000; USDA-FSIS, 2016).

Several modifications have been made since some of the early protocols for microbial testing of processed birds were established. Some of these have been relatively subtle changes that have had a considerable impact on the poultry industry. For example, a key development was the realization that antimicrobial interventions, when applied to the processing line, could in fact carryover into the diluted solutions leading to reductions in recoverable pathogens such as *Salmonella,* resulting in an underestimation of pathogen levels or even false negatives (Gamble et al., 2016). This discovery led to the development of neutralizing agents used as additives in the dilution solutions to overcome the potential inhibitory activity towards pathogens caused by carryover, ultimately leading to a more representative assessment of the true levels of *Salmonella* (Gamble et al., 2017). Since the initial research studies, the neutralized buffered diluent approach has become standardized by FSIS (2016) and has impacted the *Salmonella* prevalence in the poultry industry (Williams et al., 2018). Other developments such as more rapid molecular quantitative methods for specific pathogens and whole genome sequencing (**WGS**) continue to advance, but widespread adoption of standardized methodologies by Federal regulatory agencies and the poultry industry remain to be optimized (Ricke et al., 2018, 2019).

**Figure 2:**
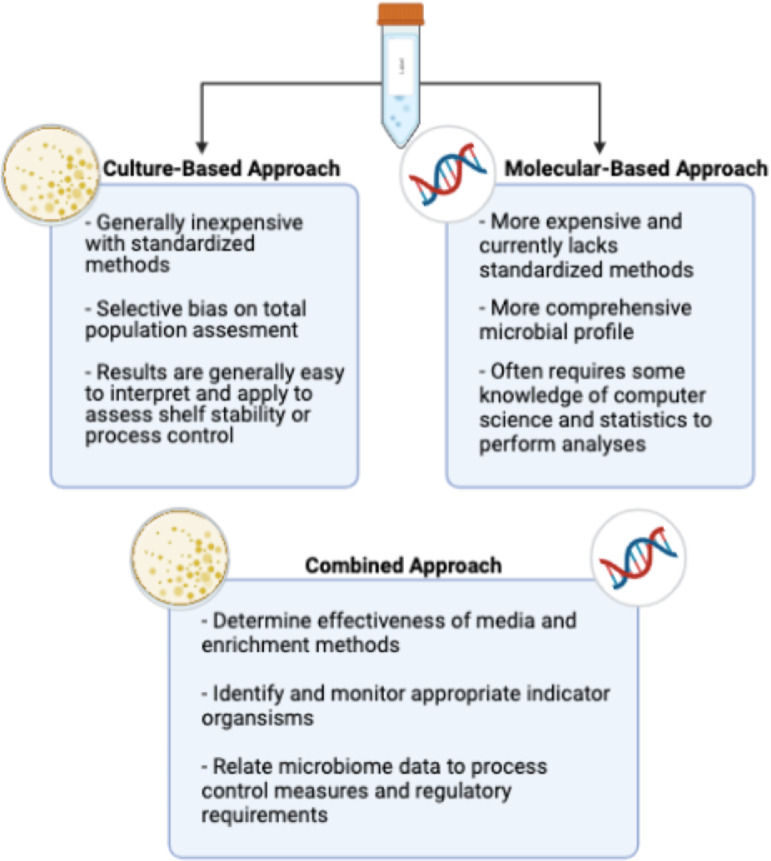
Brief summary of the advantages and disadvantages associated with culture-based and molecular-based approaches, as well as the potential applications for a combined approach. Figure created with Biorender.com.

While developments for pathogen recovery, identification, and enumeration continue to progress for poultry processing, nonpathogen methodologies have not evolved at the same pace. Reliance on media such as APC remains the standard to enumerate total bacteria levels on carcasses and, in turn, are used as a means for establishing biomapping baselines for overall microbial contamination. Likewise, the use of certain nonpathogenic indicator microorganisms, which possess behavioral traits similar to the less frequently occuring pathogens that they are expected to mimic, have been used as routine process control measures for assessing antimicrobial efficacy. However, these culture-based approaches have inherent limitations both in their ability to accurately recover viable microorganisms and their ability to clearly represent the microbial ecology of the poultry carcass surface, let alone the subsurface underneath the carcass skin regions. For example, APC media is somewhat selective since the media composition and incubation conditions would presumably favor aerobic microorganisms with a tolerance to atmospheric oxygen. However, less aerotolerant microorganisms may not be recoverable under these conditions yet could still be important from a microbial ecology and shelf-life standpoint. More comprehensive characterization of the microbial communities present on poultry carcasses are needed to achieve identification of the individuals within these microbial populations (Figure 2). Microbiome sequencing based on 16S rDNA offers a means to achieve a more complete microbial profile without being dependent on the inherent limitations of culture-based methodologies (Figure 2). The concepts of microbiome analyses and specific applications for poultry processing microbiology are discussed in the following sections.

## Microbiome Methodology and Analyses – Fundamental Concepts

Identification of individual members within microbial consortia and comparisons among either different microbial ecosystems or the same microbial ecosystem being exposed to various external conditions, such as antimicrobial treatments, has always been a challenge with conventional culture-based approaches. Certainly, some microbial activities such as changes in short-chain fatty acid (**SCFA**) production have been useful for assessing the impact of dietary shifts on the gastrointestinal tract (**GIT**) microbiota or examining the influence of SCFA on individual microorganisms such as specific pathogens (Ricke, 2003; Dittoe et al., 2018). However, relating this to all individual members of the GIT microbial community was a challenge. As molecular methods evolved, approaches such as denatured gradient gel electrophoresis (**DGGE**) offered an approach for detecting shifts in microbial populations and, depending on the methodology, the ability to identify individual members in microbial samples via sequencing of individual bands on the respective DGGE gel (Yu and Morrison, 2004; Escobar-Zapeda et al., 2015; Ricke et al., 2017). In the past few years, advances in sequencing technology and the development of high-throughput sequencers have revolutionized the ability to identify and classify microbial taxa in microbial communities based on 16S rDNA sequencing. This, along with continuous refinements in bioinformatic pipeline platforms, have provided the tools to directly compare diverse microbial populations based on the identification of individual members of the respective microbial communities.

A detailed explanation of definitions, terminology, and steps involved in 16S rDNA-based microbiome sequencing has been described previously (Amato, 2017; Ricke et al., 2017). Therefore, only an overview will be discussed in the current review. Essentially the steps to microbiome assessment of a microbial community include DNA extraction, sequencing, and conversion of raw sequencing data into bioinformatic information for taxa identification, as well as statistical comparisons of microbial diversity. Unlike WGS, microbiome sequencing is exclusively focused on the 16S rDNA gene that is present and conserved in all bacteria but still contains variable regions (V1 through V9) within the gene that are unique to individual bacteria (Robinson et al., 2016; Amato, 2017; Ricke et al., 2017). Since short read sequencing does not cover the entire 16S rDNA gene, single variable regions are targeted. Choosing a target region is a critical step as not all regions amplify equally or are sufficiently representative of all taxa (Yu and Morrison, 2004; Chakravorty et al., 2007; Comeau et al., 2017; Ricke et al., 2017).

The bioinformatics used for raw sequence analyses involves computations and programs that convert raw sequencing data into an organized biological interpretable form (Luscombe et al., 2001). Microbiome bioinformatics relies on developed software programs or analytical pipelines, such as Quantitative Insights into Microbial Ecology (**QIIME**) and mothur, to assemble and organize the sequencing data using various algorithms that are available for assembling and organizing data into graphic and numeric diversity comparisons (Schloss et al., 2009; Schloss, 2010; Gonzalez and Knight, 2012; Huse et al., 2014; Nilakanta et al., 2014; Boylen et al., 2019). Bioinformatic tools can be applied to further process the data into defined taxonomic assignments via selection of operational taxonomic units (**OTU**s) and classification into phyla, class, order, family, genus, and species based on available sequence databases (DeSantis et al., 2006; Schloss, 2010; Kozich et al., 2013; Jovel et al., 2016; Robinson et al., 2016; Amato, 2017). Of these taxonomic groups, species are the most difficult to align with published classification databases and thus are left as “OTUs” with no organism specifically named (Amato, 2017). In more recent years amplicon sequence variants (**ASV**s) have emerged as a preferred method to analyze marker-gene data rather than OTUs (Callahan et al., 2017). ASVs have the ability to differentiate samples down to the single-nucleotide level giving them a greater degree of sensitivity and specificity (Callahan et al., 2016).

When comparing microbiomes from different samples, statistical assessment of diversity levels is required and essentially involves 2 types of diversity assessments: alpha and beta diversity comparisons. Taxonomic diversity, or the number of genetically distinct microbial taxonomic OTUs within a sample, is defined as alpha diversity and involves the designation of a diversity index of each sample or treatment for comparative purposes (Ricke et al., 2017). In short, this represents a measure of microbial community complexity within a given sample. When microbiomes from distinct biological sources such as treatments are compared, the statistical assessment of the presence or absence of OTUs analyses among treatments can be done with beta diversity analyses (Robinson et al., 2016). This can be used to determine how different or similar the respective microbiomes are with each other (Robinson et al., 2016). Being able to make these comparisons across separate microbiome populations allows for conclusions regarding the impact of specific treatments of interest on the microbiome of a particular microbial ecosystem vs. other treatments and/or the control group without treatment.

## Microbiome Applications for Poultry Processing – General Concepts

Applications of microbiome analyses for poultry processing have been a relatively recent development. Most research studies have focused on a few individual steps in the processing operation, with data being generated from either commercial operations or pilot processing plants. The objectives of these studies have been varied, and therefore it is difficult to draw any overall conclusions or discuss general concepts. Consequently, there is minimal standardization or centralized protocols either for methodology or analyses beyond the general approaches used for microbiome analyses of nonpoultry processing samples. There are several reasons for this. First of all, it still remains a fundamental issue as to how microbiome methodology and bioinformatics will be used for practical poultry processing applications. As a result, there is still research being conducted that appears to be in search of a question to be addressed. Second, it is not clear how microbiome data aligns with more conventional microbial data that is based on standard culture techniques such as the presence of foodborne pathogens or general bacterial loads on carcasses. These comparisons are essential if microbiome data generated from processing plants are to be related to current regulatory requirements and/or process control measures. Finally, standard protocols for the number of representative samples to be taken, optimal locations for microbiome sampling in the processing line, and sample preparation for sequencing remain somewhat incomplete.

There have been recent attempts to standardize 16S rDNA microbiome sequencing protocols for specific applications in poultry processing. These have been described in detail previously and will only be briefly discussed in the remainder of the current review, where appropriate, when individual studies are being compared (Feye and Ricke, 2019; Feye et al., 2020a). In general, factors to be considered for protocol standardization include methods that recover DNA from processing samples with sufficient purity and quantity for optimal sequencing results, standard library preparation, choice of the hypervariable region on the 16S rDNA gene for amplification, type of sequencing platform used, and choice of bioinformatic platform (Feye et al., 2020a). Feye and Ricke (2019) have provided a detailed protocol that outlines individual steps for collecting, preparing, and sequencing poultry processing samples along with the description of the equipment needed, choice of commercial kits for DNA processing, and bioinformatics required for data analyses and interpretation. However, a note of caution that remains relevant is that much of the protocols presented currently will become out of date as sequencing technology and bioinformatics inevitably advance. For example, as long read sequencing technology improves the potential for sequencing the entire hypervariable region of the 16S rDNA, this may very well eliminate the need to choose only one of the hypervariable regions due to the limitations of short read sequencing platforms (Ricke et al., 2017). Likewise, bioinformatic platforms such as QIIME continue to add new features along with more advanced statistical tools for comprehensive data comparisons.

Despite microbiome analyses being still in its infancy for poultry processing, several potential applications have emerged that may offer the opportunity for eventual routine adoption. Certainly, conducting microbiome profiling of the microbial communities that are prominent at the various stages of processing has value. Identifying groups of microorganisms that are distinctly associated with certain steps could be useful for monitoring overall process control. For example, identification of specific groups of microorganisms before and after antimicrobial interventions are applied could be used to evaluate the effectiveness of a particular antimicrobial. This also may be useful for predicting whether shelf-life is compromised or improved depending on the antimicrobial being utilized. In addition, a more comprehensive identification of the microbial communities located on poultry carcasses may lead to the detection of specific candidate microorganisms that can be used as non-pathogen indicator microorganisms. Monitoring these indicator organisms may allow for assessing potential impact of interventions, and other process steps, on the pathogens that are at sufficiently low population levels to escape detection. Finally, microbiome sequencing of enriched microbial cultures and selective plating for pathogens can be used to assess the specificity of these methods and determine how representative they are of the microorganisms they are being used to detect and/or enumerate. The following sections illustrate some of these approaches and potential next steps for further research and development.

## Microbiome Analyses for Tracking Poultry Processing Microbial Contamination Sources

Several studies have focused on changes in the poultry carcass microbiome as the birds traverse through the various stages of poultry processing. Oakley et al. (2013) conducted some of the first high-throughput sequencing for both live bird as well as processing using 454 pyrosequencing and Illumina HiSeq platforms and the mothur pipeline to characterize taxa at each stage of poultry production. In addition to farm samples of fecal and dry and wet litter, retail and carcass rinsates were collected from the chlorinated chill tank. When comparing fecal microbiomes with the rinsates from processing, Oakley et al. (2013) noted that the richness of the rinsate microbiomes were 2 to 4 times less than those recovered from the fecal samples, but after 48 h did yield the highest proportion of unique taxa. However, these results only represent an endpoint microbial profile of processing as the steps prior to the chiller were not represented. In a pilot plant study that simulated the individual processing steps in a commercial poultry facility, Kim et al. (2017) compared microbiota populations from individual carcass rinsates at each processing step using an Illumina MiSeq platform and QIIME pipeline. Based on microbiome analyses, Kim et al. (2017) concluded that the Proteobacteria phylum decreased in the carcass rinsate solutions as birds moved along the processing line and Firmicutes increased proportionally in an almost inverse stepwise fashion.

While general microbial ecology poultry processing studies based on microbiome profiles have revealed some general transition patterns and shifts in microbial communities during processing, more specific identification of where shifts are occurring and contributing factors are possible with studies targeting specific processing steps or carcass features. For example, Rothrock, Jr. et al. (2016) demonstrated that relative abundances of both preharvest and processing microbiota increased in scalder and chiller tank waters during the day. Poultry processing microbial contamination may be influenced by carcass topology as well. Yu et al. (2020) examined bacterial composition on Belgian broilers held in retail markets using a combination of an Illumina HiSeq2500 platform-based 16S microbiome sequencing and matrix-assisted laser desorption ionization time-of-flight mass spectrum (**MALDI-TOF MS**). In addition to enumeration on aerobic and anaerobically incubated plates, they specifically focused on the bacterial populations located on the neck, back, and breast skin of conventional and organic broiler carcasses using skin homogenates. Colonies were pooled from APC plates for microbiome sequencing and individual colonies were selected from the various plates for MALDI-TOF MS determination. From these combined approaches they observed more detectable changes in microbial profiles on the neck compared to breast and back skin. The neck skin also exhibited the greatest contamination of aerobic and anaerobic bacteria followed by the back skin, and lastly the breast skin. They concluded that the neck and back represented greater risk for spoilage, and location on the carcass could be linked to microbial contamination occurring during slaughter.

Given the propensity for chicken skin to be a source of potential spoilage microorganisms as suggested by Yu et al. (2020) as well as pathogens such as *Salmonella* there is merit in exploring the microbial ecology at the tissue level (Lillard, 1989). The combination of a skin layer and overall irregular topography of a chicken carcass surface offers a wide range of niches including the feather follicles. To demonstrate the potential for follicles to be a source of bacterial contamination, Zhang et al. (2020), collected skin and follicle samples from carcasses throughout a poultry processing facility. Follicles were removed from 5 different locations on the chicken carcasses for microbial analyses and microstructural characterization. Apparently, the chicken skin follicles become a closed cavity during processing. Sequencing results of the follicles revealed that the predominant phyla in the feather follicles were Proteobacteria and Firmicutes as has been seen in other poultry processing microbiome studies. However, processing did impact the follicle microbial genera composition with *Acinetobacter* having the highest abundance, followed by *Psychrobacter, Macrococcus*, and *Comamonas* being the only other groups above 2% relative abundance after chilling. These results differed from the initial bacterial composition at the beginning of processing with *Macrococcus* decreasing, while *Acinetobacter* and *Psychrobacter* proportionally increasing as members of the follicle microbial community when processing continued. Collectively from the follicle and processing sequencing the authors concluded that follicles became contaminated by fecal material during certain stages such as evisceration, defeathering and chilling. They also suggested that considerable cross-contamination of the feather follicles occurred during chilling. A key point made by the authors is the reservoir role that follicles appear to play in retaining microbial contaminants during processing. This becomes a concern not only for retention of spoilage causing microorganisms but could contribute to the difficulty in reducing pathogen levels on carcasses.

## Microbiome Diversity Comparisons for Evaluating Poultry Processing Operations

Microbiome analyses have also proven to be insightful not only for comparing microbial ecology responses at different steps within individual processing plants, but also has utility for comparing different types of operations, sources of birds, and changes in process equipment. Factors such as age and type of bird when slaughtered would be expected to alter carcass microbial diversity (Handley et al., 2018; Wages et al., 2019). Bird rearing conditions could also be a factor. For example, when Yu et al. (2020) examined the microbiome profiles of conventionally raised birds vs. organically raised birds they concluded that rearing conditions may have more impact on carcass microbial composition than the processing plant. They noted that the carcasses originating from organically reared birds exhibited a richer microbial composition than their conventional counterparts. When the taxa were further analyzed several characteristics were observed. First, the microbial composition appeared to be unique to the organically raised birds even when shifts in microbial communities occurred upon pre-cultivation. Secondly, the aerobic bacterial populations were greater on birds from the organic poultry farms, whereas *Pseudomonas* spp., based on presumptive identification, were greater on carcasses from conventional poultry farms. The authors suggested that increased diversity on poultry carcasses from organically raised birds may be reflective of the increased exposure that such birds have with the outdoor environment. There is evidence for this diverse outdoor environmental influence from several studies of the GIT microbiome composition of pasture-raised birds that would tend to support this conceptionally (Shi et al., 2019; Ricke and Rothrock Jr., 2020). It would be of interest to conduct extensive longitudinal studies to determine the linkage between birds raised under these conditions and corresponding microbiome taxa appearing on the carcasses of these same birds.

Changes in processing equipment can also impact carcass microbial composition. At the end of poultry processing the resulting carcasses are chilled to limit pathogen growth and retain a safe product before transport (James et al., 2006). Decreasing temperature is accomplished either by immersion water chilling or air (dry) chilling of the carcasses (James et al., 2006). Some differences in meat quality have been noted between the 2 chilling systems, but the microbial differences based on bacterial culture methods have been less conclusive (Sanchez et al., 2002; James et al., 2006; Berrang et al., 2008; Carroll and Alvarado, 2008; Zhang et al., 2011; Belk et al, 2021). As pointed out previously, water chilling can be a source of microbial cross-contamination and bacteria can accumulate in chill tanks over time (Rothrock, Jr. et al., 2016; Zhang et al., 2020; Chen et al., 2020a,b). More in-depth comparisons between the 2 chilling systems are warranted to determine if chilling system does influence microbial contamination. In general, chilling systems have been shown to select for microbiome populations that are more likely cold tolerant and/or associated with spoilage (Handley et al., 2018; Chen et al., 2020a,b; Belk et al., 2021; Marmion et al., 2021).

Given the predictions that the large amounts of fresh water used for current processing plants may become a more expensive element for high volume operations as freshwater resource availability and wastewater issues come into question, there are some attractions to considering air chilling over water immersion chilling (Micciche et al., 2018; Semedo and Song, 2020; Belk et al., 2021). There have been a few studies that have directly compared air chilling with water immersion chilling using microbiome analyses. In an Australian study, Chen et al. (2020a) compared the bacterial diversity of poultry carcass microbial populations from samples collected at: (1) before scalding; (2) after scalding; (3) before immersion; chilling; (4) after immersion chilling; and after air chilling. Along with plating, microbiome samples were removed for Illumina MiSeq sequencing and the resulting analyzed via the mothur pipeline. When the 6 processing sampling sites were compared as a function of microbiome diversity Chen et al. (2020a) were able to delineate a clean vs. dirty zone in the processing line with the separation occurring at the washing step between evisceration and immersion chilling. One of the advantages of this study was the presence of both a water immersion chilling apparatus, as well as air chilling capability in the same processing plant. When the microbiome populations were compared, the air chill microbial communities were distinctly different than those recovered from any other sample site and exhibited increases in both alpha and beta diversity. In a further study by Chen et al. (2020b), that included a 12-day shelf-life time point, they found that both water immersion chilling and air chilling experienced microbial cross-contamination of the carcasses and that potential environmental contamination from the chiller walls could occur during air chilling.

The advantage of water conservation that air chilling offers remains an intriguing economic consideration. To address this, Belk et al. (2021) examined the influence of air chilled vs. water chilled on poultry processing by a combination of microbiome analyses, meat quality, shelf life, and economic assessment. Belk et al. (2021) exposed chicken carcasses to either water or air chilling followed by conversion to either bone-in or boneless breasts for further dark storage over a 7- or 14- day time periods. Carcasses that were water chilled exhibited increased weights, but air chilled carcasses had more redness and yellow on the Hunter scale. Most other quality traits such as nutrient content was not impacted. The microbial responses were influenced by chilling method. Prior to producing chicken breasts, air chilled carcass harbored more psychrotrophic microorganisms initially, but by the end of the 14-day storage period chicken breasts from both chilling methods had reached shelf-life expiration with equally high numbers of microbial contaminants. Phylogenetic diversity within each treatment was similar between the 2 chilling systems, but beta diversity comparisons did reveal cluster separation between the 2 treatments with the greatest differences observed on day 7 of dark storage. During storage, primary spoilage species belonging to *Pseudomonas* became predominant in water chilled chicken samples earlier than in air chilled samples and accounted for most of the diversity differences between the 2 chilling methods. When economic inputs such as electricity and water use are considered, air chilling appears to require less gross energy with fluctuations likely due to variations in cost of water. Taking these results collectively, Belk et al. (2021) concluded that air chilling may offer advantages in delaying spoilage and be more economically beneficial in locations with scarce water sources. However, as the authors pointed out, this will need to be explored under commercial settings. This offers the opportunity to examine these factors using greater numbers of birds in the presence of commercial poultry processing plant conditions, as well as variables such as different interventions and plant sanitation programs.

## Microbiome Taxonomy: Indicator Microorganisms and Culture Media Evaluation

The general status of microbial contamination on food and meat products is critical not just from the standpoint of the presence of pathogens as a public health concern, but spoilage potential as well. Consequently, the ability of identifying microorganisms that represent hygienic status and allow an immediate indication of microbial contamination levels is of interest. Indicator microorganisms have been defined as those which represent the general microbiological status of a food or environment while an index microorganism more specifically suggests a possible public health issue due to the potential presence of a pathogen that is ecologically similar (Chapin et al., 2014; Martin et al., 2016). In the meat industry, hygienic indicator bacteria such as total aerobic bacteria (based on APC enumeration), coliforms, and *Enterobacteriaceae* have all at one time or another been employed for assessment of quality and safety (Hutchison et al., 2006; Sofos et al., 2013; Handley et al., 2015; Blevins et al., 2017). However, most of these microorganisms have been shown to be limited in their utility as indicators. For example, most probable number quantitation of total aerobic bacterial populations on poultry carcasses was not sufficient for predicting *Salmonella* and *Campylobacter* levels (Cason et al., 1997; Blevins et al., 2017). Enumeration of aerobic bacteria, *Enterobacteriaceae*, and *Pseudomonas* in a poultry processing operation led Hutchison et al. (2006) to determine that there was only a weak relationship between presumptive hygienic processing bacterial indicator numbers and process hygiene. However, further characterization of overall microbial communities via microbiome analyses may be needed to assess the relationship between microbial contamination and hygienic status.

Given the ability to use microbiome sequencing to identify the taxonomic profiles of poultry processing samples, the concept of an “indicator” microorganism may need to be revisited. Part of the difficulty on relying on a few microorganisms from culture-based enumeration is that these microorganisms may only represent a fraction of the total number of organisms potentially present on a carcass or in the processing environment. In addition, this microbial population is likely diverse with a wide range of physiological characteristics ranging from strictly aerobic bacteria to groups of organisms with decreasing tolerance to the presence of oxygen. Consequently, regardless of the culture method used, some selection bias will occur due to culture conditions, selective agents added, and the nutrients present in the media. Molecular based methods such as PCR assays offer means to overcome some of the culture-based microbial growth steps, but because they target genes of specific microorganisms still suffer from potentially being too specific (Blevins et al., 2017). While microbiome sequencing-based approaches are not quantitative, they do provide a much more complete identification of the members of the microbial community present on a poultry carcass.

As a hygiene indicator, microbiome taxonomic characterization can potentially be applied in different ways. The taxonomy profiles resulting from microbiome sequencing can certainly be used to identify individual members of the carcass microbial consortia that might be most representative of the hygienic status. Handley et al. (2018) collected samples from 3 commercial poultry processing facilities at 3 sites, rehang, prechill, and postchill, for 16S rDNA microbiome sequencing to characterize the microbial communities at each of these stages and identify potential indicator microorganisms. As the carcasses progressed through these stages, microbial populations, estimated from plate counts, and the microbial diversity, based on microbiome analyses, declined. When taxonomy comparisons were assessed, 7 OTUs at the Family or Genus level occurred at all poultry processing plants and sampling sites, with *Pseudomonas* and *Enterobacteriaceae* being proportionally above at least 2% relative abundance through all 3 stages of processing. However, *Pseudomonas* was substantially more dominant and consistently increased from 46% to 72% at each plant from rehang to post-chill leading Handley et al. (2018) to postulate that it could be a candidate as an indicator microorganism. Wages et al. (2019) also detected *Pseudomonas*, as well as *Chryseobacterium,* from postscalder and postpicker stages from 3 different commercial processing plants.

Even though Hutchison et al. (2006) noted a limited relationship between *Pseudomonas* and process hygiene, there are several factors that may impact the presence and levels of *Pseudomonas* in processing plants and its utility as a microbial indicator candidate. Yu et al. (2020) detected greater levels of *Pseudomonas* in carcasses originating from conventionally raised birds vs. those from an organic production system. Likewise, chilling environment may be important as Chen et al. (2020a) observed a major transition to dominance by *Pseudomonas* in air chilled carcasses. In their shelf-life study, Chen et al. (2020b) did not detect *Pseudomonas* on air chilled carcasses but did find it on the walls of the air chiller and by the end of the 12 days of storage it predominated the microbiota of the packaged carcasses. Belk et al. (2021) suggested that the greater microbial diversity associated with air chilling may delay the emergence of *Pseudomonas* on poultry carcasses. In addition, they pointed out that there is a range of *Pseudomonas* species potentially present on poultry carcasses. This is consistent with previous reports that demonstrated that *Pseudomonas* populations differ when fresh poultry are compared with frozen poultry and exhibit extensive phenotypic and genotypic variability from spoiled poultry fillets (Arnaut-Rollier et al., 1999; Morales et al., 2016). Certainly, there is attraction to further refining *Pseudomonas* and developing a panel of molecular assays that could delineate the various *Pseudomonas* species and potentially use these individual species as indicator assays for predicting spoilage, but a different strategy might be to further characterize the microbiota associated with processing and use microbiome taxa information to identify representative microbial consortia for different phases of processing. The microbiome community at large might also be used as some form of a signature or core microbiome that is indicative of the microbial hygienic status of a particular stage of poultry processing. There are some examples of possibilities such as the concept of a “dirty” microbiome vs. “clean” zone for poultry processing microbiomes and/or the pyschrotroph microbiome that aligns with spoilage (Handley et al., 2018; Chen et al., 2020a; Belk et al., 2021; Marmion et al., 2021). As more microbiome data are generated across more processing locations and types of operations, the potential to refine these microbiome populations to biologically relevant signature microbiomes may be possible through meta-analyses that identifies the key factors that contribute to their diversity and taxonomic composition.

Taxonomic application of microbiome sequencing has also proved useful for evaluating culture methodology. It has been applied for determining how effective certain media are for selecting or enriching for pathogens present in food matrices. Oakley et al. (2012) applied 16S pyrosequencing to compare levels of *Campylobacter* present in poultry feces with selective media to determine the selectivity of the media and compare several selective media routinely used for *Campylobacter* selection. When comparing fecal *Campylobacter* levels of less than 4% of total sequences, selective media consisted of anywhere from 88% to 97% of the total sequences in the respective media, but incubation temperature impacted selectivity with more non-*Campylobacter* OTUs at 42°C compared to 37°C. In a more recent study, Kim et al. (2019) compared Bolton and Preston *Campylobacter* selective enrichment media for isolation of *Campylobacter* from poultry carcass rinsates. Based on 16S microbiome sequencing they detected different microbial compositional profiles for the 2 media, with only 31.57% in common between the 2 and only a minimal proportion of the OTUs being identified as *Campylobacter*. However, the proportion of *Campylobacter* was greatly increased if the selective enrichment was followed by plating on selective media but depended on the type of selective plate media. As the authors suggested microbiome analyses may provide a means to optimize the sequence of selective media chosen for enumerating pathogens such as *Campylobacter* from poultry processing samples. Kim et al. (2017) pursued a different approach to evaluating selective media for poultry processing by pooling colonies from a *Campylobacter* selective medium and demonstrated that less than half on average were identified as *Campylobacter*. Furthermore, when pooled colonies were sequenced from APC petrifilm different microbial populations were detected at various stages of poultry processing and before and after application of interventions.

## Conclusions

Studies involving microbiome sequencing for poultry production and processing have increased dramatically in the past few years as methods have become standardized and availability of sequencing platforms are more economical for routine analyses. However, studies still for the most part focus on characterization of the microbial ecology of poultry processing. While these studies certainly offer insight into identifying factors that may be important to consider for assessing poultry processing microbiology, the commercial applications that have practical deliverables for immediate use, still for the most part, remain to be identified. The basic bioinformatic information gleaned from microbiome 16S sequencing are essentially taxonomy identification and diversity assessment of microbial communities. As discussed in the current review, both of these metrics have practical applications for poultry processing microbiology. Diversity comparisons offer the opportunity to not only compare stages of processing within a plant but changes over time, flock-to-flock, and between sanitation cycles. In addition, plant-to-plant variation can be assessed as a function of geographical sites and other contributing factors. At first glance, the practicality of these types of information may not be obvious. However, as this data is collected over time and compiled, it is anticipated that large sets of such data could be further analyzed, baselines established, and conclusions drawn that will lead to more precise microbial tracking and source attribution. This type of information may be utilized for identifying problematic factors associated with sanitizing as well as processing interventions. Likewise, the taxonomy data as discussed has immediate utility for identifying either individual or community groups of potential indicator microorganisms for hygienic monitoring. A more novel application may be the evaluation of the relative effectiveness of selective media and using this information to identify which media are optimal for a specific pathogen.

An important consideration that needs to accompany any strategy for making microbiome analyses more commercially viable is the development of educational and training materials that render both the microbiome sequencing technology and the associated bioinformatics more approachable by lay audiences. This does not just refer to the technical methodologies associated with microbiome analyses, but an appreciation for the complexity of the data and the need to make that data cybersecure. This requires awareness of several aspects of not only the processing of complex data sets, but the subsequent interpretation for practical purposes. As the statistical power increases and modeling predictions become more feasible for a variety of applications including microbial hygiene patterns in poultry processing, data management and security will be an increasing concern. Therefore, Thompson et al. (2017) have pointed out that cybersecurity infrastructure in the food and poultry industries will become a critical issue for education and training. When Feye et al. (2020b) surveyed food science students for background in computer. technology and cybersecurity topics, they concluded that students were relatively deficient in these skills Feye et al. (2020b) suggested that more specialized coursework needed to be devoted to not only the technological aspects of topics such as microbiome sequencing, but also data management and security to appropriately train the next generation of food and poultry science professionals and workforce members. This holds true for current poultry and allied industry personnel as well. The development of practical workshops that explain the basics of microbiome data generation and interpretation, as well as cybersecurity awareness, are needed if bioinformatics is to become a practical and routine tool for the poultry industry.

## Disclosures

There is no conflict of interest with any of the authors.
